# Unheard Victims: Multidisciplinary Incidence and Reporting of Violence in an Emergency Department

**DOI:** 10.5811/westjem.2021.2.50046

**Published:** 2021-05-07

**Authors:** Sarayna S. McGuire, Aidan F. Mullan, Casey M. Clements

**Affiliations:** * Mayo Clinic, Department of Emergency Medicine, Rochester, Minnesota; † Mayo Clinic, Department of Quantitative Health Sciences, Rochester, Minnesota

## Abstract

**Introduction:**

Workplace violence in the emergency department (ED) is a serious threat to staff and is likely to go unreported. We sought to identify the incidence of violence among staff at our academic ED over a six-month period.

**Methods:**

An anonymous survey was sent to all ED staff, asking whether respondents had experienced verbal abuse or physical assault over the prior six months and whether they had reported it. Those working in the department <6 months were excluded from analysis. We used chi-squared comparison to analyze the results.

**Results:**

We analyzed 242 responses. Overall, 208 (86%) respondents indicated being verbally abused in the preceding six months, and 90 (37%) indicated being physically assaulted. Security officers had the highest incidence of verbal abuse (98%), followed by nursing (95%), patient care assistants (PCA) (90%) and clinicians (90%), phlebotomists (75%), care team assistants (73%), registration staff (50%) and electrocardiogram (ECG)/radiology technicians (50%). Security also had the highest incidence of physical assault (73%), followed by nursing (49%), PCAs (30%), clinicians (24%), phlebotomists (17%), and ECG/radiology technicians (13%). A total of 140 (69%) non-security personnel indicated that they never report incidents of violence.

**Conclusion:**

Our results indicate that violence in the ED affects more than just nurses and doctors. As health systems seek to improve the safety of their employees in violence-prone areas, it is imperative that they direct initiatives to the entire healthcare team as no one group is immune.

## INTRODUCTION

### Background

Workplace violence in healthcare is a serious threat to staff. Between 2011–2013, the number of workplace assaults averaged approximately 24,000 annually, with nearly 75% occurring in healthcare settings. Data from the Bureau of Labor Statistics show that incidents of serious workplace violence were four times more common among healthcare workers than those in private industry.^1^ Emergency departments (ED) and psychiatric hospitals are two areas in healthcare where violence is most commonly reported.

### Importance

There is a concerning rise in ED violence, with one in five ED directors reporting guns or knives brought into the ED on a daily or weekly basis.^2^ Violence against healthcare workers continues to make the news, and hospital-based shootings nearly doubled between 2000–2011.^3^ Among ED nurses, prior research has shown an annual incidence of verbal and physical abuse ranging from 39–98% and 13–67%, respectively.^4^,^5^ Among emergency physicians, the incidence has ranged from 75%–96% and 51%–78%, respectively.^1^,^4^,^5^ However, this likely represents under-reported data as only 30% of nurses and 26% of physicians go on to report incidents of violence.^6^ According to a 2018 poll from the American College of Emergency Physicians, nearly 70% of respondents believed that violence in the ED has increased during the previous five years and nearly 80% felt that patient care was affected as a result.^7^

### Goals of This Intervention

Exposure to workplace violence impacts the entire team; however, there is a paucity of research evaluating the incidence of violence experienced by the ED multidisciplinary care team and how it compares to institutional reporting. We sought to survey all staff at our academic ED to identify the incidence of verbal abuse and physical assault over a six-month period and compare responses to documented incident reports from the same time period to evaluate for under-reporting of violence. We also sought to obtain baseline characteristics of respondents to evaluate for risk factors for violence or under-reporting. We hypothesized that nearly all members of the ED multidisciplinary care team have been exposed to verbal abuse over a six-month time period, with many of these incidents going under-reported, and that a significant percentage of staff have also experienced physical assault during the same time frame.

## METHODS

### Study Design and Setting

This descriptive prospective study took place between April–May 2020 within the ED of a large, academic, Level 1 trauma center in a small urban city in the Midwest. The ED sees an average of 78,000 patients annually and has 24/7 security presence available.

### Selection of Participants

The target population consisted of all ED staff, including clinicians (attending and resident physicians, and advanced practice providers [APP]), nursing, care team assistants (CTA) who provide clerical support and limited patient interaction, patient care assistants (PCA), electrocardiogram (ECG) and radiology technicians, phlebotomists, registration staff, and security officers who worked in the ED at least six months prior to taking the survey. After review by the institutional review board (IRB), the survey (described below) was emailed to all distribution lists for the abovementioned target population with a cover letter describing the study purpose, directions for participation, and information regarding informed consent. The questionnaire included a statement of informed consent at the beginning, and completion indicated participant consent for inclusion in the study. Two reminder notices were sent two weeks apart through the same method. The IRB reviewed this study and materials and deemed it exempt from approval requirement.

### Measurements

We developed an anonymous REDCap survey (Research Electronic Data Capture, Vanderbilt University, Nashville, TN)^8^ that included both multiple-choice and Likert-scale response questions. This survey was distributed broadly by department and job type to anyone who might work in the ED, even occasionally. Respondents were asked to self-select for if they had done any work in an ED in the preceding six months. Participants were asked to indicate whether they had experienced any of the following forms of verbal abuse in the prior six months (October/November 2019–April/May 2020) while working in the ED: threatening tone of voice; abusive language/statement; harassment (eg, racial, gender, sexual); or personal verbal threats (eg, threat of physical or sexual violence, threat of physical assault to occur outside the workplace). Participants were asked to indicate whether they had experienced any of the following forms of physical assault in the prior six months while working in the ED: physical assault with weapons (including hospital equipment); physical assault with bodily fluids (eg, saliva, urine, feces, wound exudate, blood, or spit); or physical assault in the form of punching, biting, rough handling, scratching, kicking, shoving/pushing, or hitting. If answering affirmatively to any of these choices, respondents were asked to indicate whether they had formally reported the incident.

**Population Health Research Capsule**

What do we already know about this issue?

*Serious workplace violence incidents are five times more common in healthcare than other industries, and events are often not reported*.

What was the research question?

*What is the incidence of workplace violence in the emergency department, risk factors, and why is it under-reported?*

What was the major finding of the study?

*Most staff, including support disciplines, experienced violence and most incidents went unreported*.

How does this improve population health?

*As health systems seek to improve the safety of their employees, they must direct initiatives to the entire healthcare team since no group is immune*.

We used Likert scales to measure participants’ perceptions of safety and estimated frequency of verbal abuse, physical assault, and reporting of incidents of workplace violence in the prior six months. Standard demographic measures were collected, including gender, profession, primary shift worked, and years of experience, and whether the employee had worked in the ED for at least six months. We asked the institution’s Office of Security to provide de-identified data on the number of verbal abuse and physical assault incident reports filed by ED staff during the same time period for comparison.

### Outcomes

The primary outcome was the incidence of verbal abuse and physical assault experienced by ED staff in a six-month time period as indicated by survey responses. The secondary outcome was the comparison of this self-revealed data to formally reported incidents during the same time period.

### Data Analysis

Survey responses were summarized with frequency counts and percentages. We performed group comparisons of survey responses using chi-squared tests. Pairwise group comparisons were performed using odds ratios calculated from frequency counts. Confidence intervals were generated using asymptotic Gaussian approximation. We converted Likert-scale responses to the perceptions of safety question to a numeric rank based on the strength of sentiment. A two-sided Wilcoxon rank-sum test and Kruskal-Wallis test were performed on these ranks to compare responses between gender and years-of-experience groups, respectively. All tests were two-sided and *P*-values less than 0.05 were considered significant. We performed analyses using R version 3.6.2 (The R Foundation for Statistical Computing, Vienna, Austria).

## RESULTS

### Characteristics of Study Subjects

A total of 261 responses were received. Seventeen respondents indicated working in the ED less than six months and two respondents indicated working in management with no clinical duties—these 19 responses were excluded from analysis. We included the 242 remaining responses in our analysis (Table 1). The cohort was 59.5% female. The most common positions were nursing staff (80/242, 33.1%), security (40/242, 16.5%), and attending physicians (28/242, 11.6%).

### Violence by Position

Overall, 208 (86%) respondents indicated they had been verbally abused in the preceding six months (Table 2). Security officers had the highest incidence of verbal abuse (98%), followed by nursing (95%), PCAs (90%) and clinicians (90%), phlebotomists (75%), CTAs (73%), registration staff (50%), and ECG/radiology technicians (50%). Non-security and non-nursing personnel indicated an incidence of verbal abuse of 78%, which was significantly lower than either security (odds ratio [OR] = 0.08, 95% confidence interval [CI], 0.01 – 0.62, *P* <.001) or nursing staff (OR = 0.17, 95% CI, 0.06 – 0.50, *P* < .001).

Staff indicated how often they were verbally abused by patients or family members in the prior six months (Table 3). Security personnel had the highest proportion of responses indicating incidents of verbal abuse at least every week (16/40, 40%), followed by nurses (30/80, 38%). For non-security and non-nurse employees, only 11% of respondents indicated verbal abuse occurring at least every week, which was significantly lower than either security (OR = 0.19, 95% CI, 0.08 – 0.45,*P* < .001) or nursing staff (OR = 0.22, 95% CI, 0.11 – 0.44, *P* < .001).

Overall, 90 (37.2%) respondents indicated that they had been physically assaulted in the preceding six months (Table 4). Security officers had the highest incidence of physical assault (73%), followed by nursing (49%), PCAs (30%), clinicians (24%), phlebotomists (17%), and ECG/radiology technicians (13%). Neither CTAs nor registration staff revealed any physical assault. Again, security had the highest frequency of assault, with 29 of 40 (73%) respondents indicating being physically assaulted at least once. Nurses had the next highest frequency of assault (39/80, 49%). For non-security and non-nurse staff, 22 (18%) respondents indicated at least one incident of physical assault. This frequency was significantly lower than security (OR = 0.08, 95% CI, 0.04 – 0.19, *P* < .001) and nursing staff (OR = 0.23, 95% CI, 0.12 – 0.44, *P* < .001).

Table 5 describes the frequency of reporting events of workplace violence, grouped by position. Security personnel had the lowest proportion of respondents indicating they never report incidents, with seven (18%) responding in this way. Comparatively, 140 (69%) non-security personnel responded that they never report incidents. The odds that a non-security staff member responded “Never” were 11 times higher than for security personnel (OR = 10.65, 95% CI, 4.47 – 25.38, *P* < .001).

### Violence by Gender

Table 6 provides the number of respondents experiencing verbal abuse, grouped by gender. Overall, there was no difference in the incidence of verbal abuse between genders (female: 85%; male: 87%, *P* = 0.70). Males were more likely to report incidents of verbal abuse compared to females (OR = 3.87, 95% CI, 1.77 – 8.47, *P* < .001). However, once we account for employee position, there was no difference in reporting between males and females. For security personnel, 16/29 (55%) males and 4/6 (67%) females indicated reporting verbal abuse experienced (OR = 1.63, *P* = 0.61). For non-security personnel, 7/53 (13%) males and 7/116 (6%) females indicated reporting the abuse (OR = 2.37, *P* = 0.12).

Table 7 summarizes the incidence of physical assault. There was no significant difference in the overall incidence of physical assault between genders (female: 33%; male: 43%, *P* = 0.16). However, males experienced 2.8 times more occurrences of assault with bodily fluids compared to females (OR = 2.82, 95% CI, 1.43 – 5.55, *P* = .002). Males who experienced physical assault were more likely to report the incident compared to females (OR = 3.79, 95% CI, 1.57 – 9.18, *P* = .003). Again, there was no difference in reporting between males and females after accounting for employee position. Among security personnel, 19/21 (90%) males and 5/6 (83%) females indicated reporting physical assault experienced (OR = 1.9, *P* = 0.63). For non-security personnel, only 6/20 (30%) males and 9/41 (22%) females indicated reporting physical assault experienced (OR = 1.52, *P* = 0.5).

### Violence by Shift

There was no difference in the overall incidence of verbal abuse between shifts (x^2^ = 4.63, *P* = .20); However, staff working during the evening reported 69% fewer instances of abusive tone (OR = 0.31, 95% CI, 0.15 – 0.64, *P* = .001). Staff working day or overnight shifts were 4.2 times more likely to report incidents of verbal abuse compared to those working evening or rotating shifts (OR = 4.17, 95% CI, 1.85 – 9.39, *P* < .001). There was no significant difference in physical assault related to shifts (x^2^ = 3.97, *P* = .26). Moreover, there was no significant difference in the number of respondents reporting incidents of assault (x^2^ = 7.01, *P* = .071).

### Violence by Years of Experience

Staff members with less than four years or more than 21 years of experience were more likely to experience some form of verbal abuse compared to staff members with 5–20 years of experience (OR = 2.94, 95% CI, 1.31 – 6.61, *P* = .007). There was no difference in the number of respondents reporting their incidents of verbal abuse between years of experience (x^2^ = 4.18, *P* = .24). There was no difference in the number of respondents indicating some form of physical assault between experience groups (x^2^ = 6.00, *P* = 0.11). Additionally, there was no difference in the number of respondents reporting physical assault between experience groups (x^2^ = 2.02, *P* = .57).

### Perceptions of Safety

When asked how safe respondents felt as a staff member working in the ED, 100% of respondents indicated subjectively feeling safe, with 11.1% indicating feeling extremely safe (27/242), 48.8% very safe (118/242), 35.5% moderately safe (86/242), and 4.5% slightly safe (11/242). No respondents indicated feeling unsafe. Responses were converted to a numeric rank based on the strength of sentiment, with “slightly safe” the lowest score at 1 and “extremely safe” the highest score at 4. Males had a higher perceived safety compared to females (*P* = .016). The average response rank for males was 2.81, compared to 2.56 for females. When evaluating for perceptions of safety among staff with different years of experience, there was no significant difference in perceived safety between the experience groups (*P* = .40).

### Official Incident Reports

During the same six-month time frame respondents were surveyed, there were only 11 official incident reports made to the Office of Security regarding verbal threats or harassment and 18 reports of physical assaults. Compared to self-reported data from the survey, this corresponds to a 5% and 18% reporting rate, respectively.

## DISCUSSION

Similar to prior research, our survey of ED staff showed a high incidence of verbal abuse (86%) and physical assault (37%) within our ED over a six-month time period. Through surveying the entire multidisciplinary team, we were able to demonstrate that all team roles experienced verbal abuse at some point in a six-month time period, and nearly all experienced physical assault with the exception of CTAs and registration staff. Even so, interestingly all 242 respondents indicated feeling some degree of safety in our ED. We recognize that this subjective reporting of safety may be misleading and may be attributed to a selection bias as healthcare employees who feel unsafe in their workplace are more likely to transfer out of the department and may have been missed by our survey. This finding may also mirror prior literature that healthcare employees are resistant to the belief that they are at risk for patient-initiated violence and experience a complacency in thinking that violence is simply “part of the job.”^1^

Security personnel were more likely to formally report incidents compared to non-security personnel victims. This may be due in part to the nature of their job and the frequency with which they experience violence, as well as familiarity with the reporting process as departmental incident reports are submitted to their office. Concerningly, 69% of non-security personnel indicated that they never report incidents of violence. This was corroborated with a review of official incident reports received during the same time period. Barriers to reporting are multifactorial and include, as described above, the belief that violence is “part of the job,” confusion over what constitutes violence, unfamiliarity with reporting processes, lack of available time at work for reporting incidents, fear of retribution from supervisors, and perceived lack of institutional support.^1^,^9^ Our study findings indicate that future efforts to increase incident reporting within the ED should focus on the entire multidisciplinary team, including visiting staff assigned to non-ED departments such as phlebotomy, cardiac monitoring (ECG), and radiology.

In terms of isolating specific risk factors, we found no difference in the overall incidence of violence between genders; however, males were significantly more likely to report incidents of both verbal and physical abuse compared to females. To our knowledge, this has not been previously described in the literature. Although we found a difference in the reporting of violence between genders, this difference was not significant once we accounted for employee position. This is likely due to a greater proportion of males in our study working in security (35% males, 5% females), and security personnel indicating a higher rate of violence reporting regardless of gender. Future studies with larger cohort sizes should seek to identify whether there is a difference in reporting between genders. Coincidentally, females had a significantly lower perceived perception of safety in our ED compared to their male counterparts. Thus, additional research should seek to more clearly establish the reasons why more females choose not to report incidents of violence.

There was no significant difference in the overall incidence of violence between shifts; however, staff working daytime or overnight shifts were more likely to report incidents of verbal abuse. This may be explained by the higher frequency of incident reporting by security staff and the fact that security officers in our institution work 12-hour shifts, considered either day shift or overnight shifts, with only non-security personnel working evening or rotating shifts. Future research should continue to distinguish what additional demographic factors may be contributing to the lack of violence reporting.

The unique environment of the ED contributes to its propensity for violence: stress among patients, families, and visitors; long wait times and delays; crowding; unrestricted 24-hour access; low socioeconomic status; substance abuse; patients with behavioral health issues; gang activity; and frequent delivery of “bad news” have all been suggested to contribute to the elevated incidence of violence.^1^,^10^ A multidisciplinary study of healthcare workers found exposure to workplace violence significantly correlated with burnout, and a separate survey of ED nurses found that 94% of those experiencing violence in the workplace exhibited symptoms of post-traumatic stress.^11^ In addition to its impact on patient care and detriment to employee wellbeing, violence has a substantial financial impact for employers and the economy. Financial costs of workplace violence include lost time/wages; medical costs of employee injury, disability, and/or death; and attrition.^12^ According to a 2017 report commissioned by the American Hospital Association, hospitals spent an estimated $1.1 billion in security and training costs to prevent violence, and an additional $429 million to cover costs such as medical care, staffing, and insurance resulting from violence against staff.^13^,^14^ Future research should attempt to characterize the mental and physical toll on the multidisciplinary ED care team to help direct efforts for employee wellbeing.

This study’s findings have important clinical implications. The incidence of verbal abuse among our multidisciplinary ED care team was nearly 6 of every 7 staff members, and yet these incidents were almost never reported to the institution. The incidence of physical assault was more than 2 of every 5 staff members and, again, the majority went unreported. Nearly 7 out of every 10 non-security staff members declined to officially report the violence they experienced. Findings from this study suggest that the pervasive nature of violence in healthcare is still underappreciated and that increased efforts are needed to protect ED staff members and support and encourage or incentivize accurate and reliable reporting.

## LIMITATIONS

This study has several important limitations. To preserve anonymity of employees, the study was sent to email distribution lists (DL) and included some DLs with employees working in other departments other than the ED (eg, phlebotomy, and ECG and radiology technicians), or who also worked at additional sites elsewhere in our health system (eg, clinicians). Thus, it is not possible to know the actual number of employees from different disciplines who work in the ED to estimate a response rate for our survey. Additionally, to further preserve anonymity, we did not ask in-depth demographic questions. Without knowing full-time vs part-time status of respondents, it is possible that some responses came from employees working part time and this may have skewed our incidence of violence. The definition of “verbal abuse” is highly subjective to individual respondents and survey inclusion of “threatening tone of voice’” may have contributed to over-reporting of verbal abuse in general by respondents.

The study was also subject to recall and reporting bias in terms of violence experienced over a six-month time period, as well as the reporting of incidents. We acknowledge that because this was a single-center study some aspects may not be generalizable to all institutions or geographic regions. However, the finding of under-reporting is not dissimilar to other published studies,^15^,^16^ and the fact that abuse and violence affect previously unstudied populations including ancillary services and clerical assistant staff is important and not likely related to local factors.

## Figures and Tables

**Table 1:** Cohort demographics of emergency department staff surveyed about workplace violence.

	Female (N = 144)	Male (N = 95)	Overall (N = 242)*
Job position			
Clinician	23 (16%)	26 (27.4%)	49 (20.2%)
Attending physician	12 (8.3%)	16 (16.8%)	28 (11.6%)
Resident physician	10 (6.9%)	8 (8.4%)	18 (7.4%)
Advanced practice provider	1 (0.7%)	2 (2.1%)	3 (1.2%)
Care team assistant	11 (7.6%)	0 (0.0%)	11 (4.5%)
Nursing	64 (44.4%)	16 (16.8%)	80 (33.1%)
Patient care assistant	8 (5.6%)	2 (2.1%)	10 (4.1%)
Phlebotomist	16 (11.1%)	8 (8.4%)	24 (9.9%)
Radiology/ECG	14 (9.7%)	10 (10.5%)	24 (9.9%)
Registration	2 (1.4%)	2 (2.1%)	4 (1.7%)
Security	6 (4.2%)	31 (32.6%)	40 (16.5%)
Primary shift			
Day	34 (23.6%)	30 (31.6%)	65 (26.9%)
Evening	31 (21.5%)	10 (10.5%)	41 (16.9%)
Night	22 (15.3%)	22 (23.2%)	46 (19.0%)
Rotating	57 (39.6%)	33 (34.7%)	90 (37.2%)
Years of experience			
0–4 Years	45 (31.2%)	30 (31.6%)	76 (31.4%)
5–10 Years	33 (22.9%)	21 (22.1%)	55 (22.7%)
11–20 Years	44 (30.6%)	27 (28.4%)	71 (29.3%)
21+ Years	22 (15.3%)	17 (17.9%)	40 (16.5%)

* 3 respondents chose not to disclose gender/sex.

*ECG*, electrocardiogram.

**Table 2:** Incidence of verbal abuse in the prior six months by position.

Position	Any abuse	Threatening tone	Abusive language	Harassment	Verbal threats	Reported abuse
Clinician	44 (90%)	42 (86%)	38 (78%)	19 (39%)	17 (44%)	1 (2%)
Attending physician	25 (89%)	23 (82%)	23 (82%)	9 (32%)	10 (4%)	1 (4%)
Resident physician	16 (89%)	16 (89%)	12 (67%)	10 (6%)	6 (33%)	0 (0%)
Advanced practice provider	3 (100%)	3 (100%)	3 (100%)	0 (0%)	1 (33%)	0 (0%)
Care team assistant	8 (73%)	7 (64%)	7 (64%)	2 (18%)	1 (9%)	1 (13%)
Nursing	76 (95%)	74 (93%)	72 (90%)	41 (51%)	44 (55%)	8 (11%)
Patient care assistant	9 (90%)	8 (80%)	9 (90%)	3 (30%)	3 (30%)	0 (0%)
Phlebotomist	18 (75%)	12 (50%)	17 (71%)	6 (25%)	2 (8%)	4 (22%)
Radiology/ECG	12 (50%)	10 (42%)	10 (42%)	3 (13%)	1 (4%)	0 (0%)
Registration	2 (50%)	2 (50%)	1 (25%)	0 (0%)	0 (0%)	0 (0%)
Security	39 (98%)	38 (95%)	38 (95%)	27 (68%)	27 (68%)	22 (56%)

Note: Reported abuse is given as the percent of respondents who indicated any abuse that reported the incident.

*ECG*, electrocardiogram.

**Table 3:** Frequency of verbal abuse in the prior six months by position.

Position	Every day or two	Every week	Every month	Less than every month
Clinician	1 (2%)	5 (10%)	14 (29%)	29 (59%)
Attending physician	1 (4%)	4 (14%)	8 (29%)	15 (54%)
Resident physician	0 (0%)	1 (6%)	4 (22%)	13 (72%)
Advanced practice provider	0 (0%)	0 (0%)	2 (67%)	1 (33%)
Care team assistant	0 (0%)	2 (18%)	3 (27%)	6 (55%)
Nursing	9 (11%)	21 (26%)	34 (42%)	16 (20%)
Patient care assistant	2 (20%)	1 (10%)	1 (10%)	6 (60%)
Phlebotomist	1 (4%)	2 (8%)	2 (8%)	19 (79%)
Radiology/ECG	0 (0%)	0 (0%)	5 (21%)	19 (79%)
Registration	0 (0%)	0 (0%)	0 (0%)	4 (100%)
Security	3 (8%)	13 (32%)	17 (42%)	7 (18%)

*ECG*, electrocardiogram.

**Table 4:** Incidence of physical assault in the prior six months by position.

Position	Any assault	Assault-weapons	Assault-fluids	Assault-body	Reported assault	Reported abuse
Clinician	12 (24%)	1 (2%)	8 (16%)	8 (16%)	2 (17%)	1 (2%)
Attending physician	7 (25%)	1 (4%)	5 (18%)	3 (11%)	1 (14%)	1 (4%)
Resident physician	4 (22%)	0 (0%)	2 (11%)	4 (22%)	0 (0%)	0 (0%)
Advanced practice provider	1 (33%)	0 (0%)	1 (33%)	1 (33%)	1 (100%)	0 (0%)
Care team assistant	0 (0%)	0 (0%)	0 (0%)	0 (0%)	N/A	1 (13%)
Nursing	39 (49%)	5 (6%)	14 (18%)	34 (43%)	12 (31%)	8 (11%)
Patient care assistant	3 (30%)	0 (0%)	1 (10%)	3 (30%)	1 (33%)	0 (0%)
Phlebotomist	4 (17%)	0 (0%)	3 (13%)	3 (13%)	1 (25%)	4 (22%)
Radiology/ECG	3 (13%)	0 (0%)	0 (0%)	3 (13%)	0 (0%)	0 (0%)
Registration	0 (0%)	0 (0%)	0 (0%)	0 (0%)	N/A	0 (0%)
Security	29 (73%)	2 (5%)	18 (45%)	28 (70%)	24 (83%)	22 (56%)

Note: Reported abuse is given as the percent of respondents who indicated any abuse and reported the incident.

*ECG*, electrocardiogram.

**Table 5:** Frequency of abuse reporting in the prior six months by position.

Position	Always	Often	Sometimes	Rarely	Never	Not applicable
Clinician	0 (0%)	1 (2%)	2 (4%)	3 (6%)	40 (82%)	3 (6%)
Attending physician	0 (0%)	1 (4%)	2 (7%)	2 (7%)	21 (75%)	2 (7%)
Resident physician	0 (0%)	0 (0%)	0 (0%)	0 (0%)	17 (94%)	1 (6%)
Advanced practice provider	0 (0%)	0 (0%)	0 (0%)	1 (33%)	2 (67%)	0 (0%)
Care team assistant	0 (0%)	0 (0%)	1 (9%)	2 (18%)	7 (64%)	1 (9%)
Nursing	3 (4%)	3 (4%)	5 (6%)	16 (20%)	50 (62%)	3 (4%)
Patient care assistant	1 (10%)	0 (0%)	1 (10%)	2 (20%)	5 (50%)	0 (0%)
Phlebotomist	1 (4%)	0 (0%)	2 (8%)	4 (17%)	15 (62%)	2 (8%)
Radiology/ECG	0 (0%)	0 (0%)	0 (0%)	1 (4%)	19 (79%)	4 (17%)
Registration	0 (0%)	0 (0%)	0 (0%)	0 (0%)	4 (100%)	0 (0%)
Security	14 (35%)	9 (22%)	6 (15%)	1 (2%)	7 (18%)	3 (8%)

*ECG*, electrocardiogram.

**Table 6:** Incidents of verbal abuse by respondent gender.

Gender	Any abuse	Threatening tone	Abusive language	Harassment	Verbal threats	Reported abuse
Female	122 (85%)	114 (79%)	110 (76%)	59 (41%)	47 (33%)	11 (9%)
Male	83 (87%)	76 (80%)	79 (83%)	40 (42%)	45 (47%)	23 (28%)
Overall	208 (86%)	193 (80%)	192 (79%)	101 (42%)	95 (39%)	36 (17%)

Note: Reported abuse is given as the percent of respondents who indicated any abuse and reported the incident.

**Table 7:** Incidents of physical assault by respondent gender.

Gender	Any assault	Assault - weapons	Assault - fluids	Assault - body	Reported assault	Reported abuse
Female	47 (33%)	5 (3%)	17 (12%)	41 (28%)	14 (30%)	11 (9%)
Male	41 (43%)	2 (2%)	26 (27%)	36 (38%)	25 (61%)	23 (28%)
Overall	90 (37%)	8 (3%)	44 (18%)	79 (33%)	40 (44%)	36 (17%)

Note: Reported abuse is given as the percent of respondents who indicated any abuse that reported the incident.
